# Association of hemoglobin-to-red cell distribution width ratio with diabetic retinopathy risk and severity

**DOI:** 10.3389/fendo.2025.1622460

**Published:** 2025-08-11

**Authors:** Bin Wang, Hui Li, Lin Wang, Zaihong Chen

**Affiliations:** ^1^ Department of Ophthalmology, Chongqing Emergency Medical Center, Chongqing, China; ^2^ Department of Ophthalmology, The Second Affiliated Hospital of Chongqing Medical University, Chongqing, China; ^3^ Department of Nursing, Sichuan Nursing Vocational College, Chengdu, Sichuan, China

**Keywords:** diabetic retinopathy (DR), hemoglobin-to-red cell distribution width ratio (HRR), inflammation, NHANES (National Health and Nutrition Examination Survey), risk stratification

## Abstract

**Background:**

Diabetic retinopathy (DR) is a leading cause of blindness in diabetic patients, driven by inflammation, oxidative stress, and hypoxia. The hemoglobin-to-red cell distribution width ratio (HRR) is a novel inflammatory marker reflecting these pathological mechanisms. This study aimed to investigated the association of HRR with DR risk and severity.

**Methods:**

Data from the 2005–2008 National Health and Nutrition Examination Survey were analyzed using weighted logistic regression, subgroup analysis, restricted cubic splines, mediation analysis, and other methods.

**Results:**

Among 1,260 diabetic patients, HRR was inversely associated with DR development (OR = 0.85, P = 0.008), remaining significant post-propensity score matching. A non-linear relationship was identified, with an inflection point at HRR = 10.81 (P for non-linearity < 0.001), above which the protective effect strengthened with increasing HRR. Mediation analyses revealed diastolic blood pressure (15.9% mediation) and HbA1c (60.5% competitive mediation) as partial mediators of the HRR-DR association. HRR was also inversely associated with DR severity, particularly proliferative DR (vs. mild non-proliferative DR: OR = 0.67, P = 0.030; vs. severe non-proliferative DR: OR = 0.04, P = 0.002).

**Conclusions:**

HRR is negatively correlated with DR onset and progression, highlighting its potential as a cost-effective biomarker for DR risk stratification.

## 1 Introduction

Diabetes mellitus poses a major global health burden, with diabetic retinopathy (DR) as a leading microvascular complication (1). DR is a primary cause of blindness among adults aged 20–74 years in developed countries, significantly impairing patients’ quality of life and increasing healthcare costs (1). The pathogenesis of DR is driven by chronic hyperglycemia, which triggers oxidative stress, inflammation, and vascular dysfunction (2). Vitreous proteomic analyses reveal elevated levels of inflammatory markers, including interleukin-1β (IL-1β), tumor necrosis factor (TNF), interleukin-6 (IL-6), interleukin-8 (IL-8), and chemokine ligand 2 (CCL2), in patients with DR (2). The interplay of chronic hyperglycemia, inflammation, and oxidative stress leads to retinal ischemia and hypoxia (2). Early detection and risk stratification of DR are essential to prevent irreversible vision loss.

The hemoglobin-to-red cell distribution width ratio (HRR) is a novel biomarker that sensitively reflects systemic inflammation, nutritional status, and overall health (3). HRR combines hemoglobin, which declines in inflammatory states due to disrupted erythropoiesis and iron metabolism (3, 4), and red cell distribution width (RDW), which rises with impaired erythrocyte maturation and oxidative stress (5). A lower HRR indicates heightened inflammation, oxidative stress, and impaired oxygen delivery, key mechanisms to diabetic complications (6). HRR outperforms its individual components as a predictor in various clinical settings (7). Growing evidence supports HRR’s prognostic value in conditions such as cardiovascular disease (6), heart failure (8), cancer (9), and stroke (10). However, its association with DR remains uninvestigated, despite HRR’s reflection of inflammation, oxidative stress, and hypoxia—key drivers of DR pathogenesis.

To address this gap, this study analyzed data from the National Health and Nutrition Examination Survey (NHANES) to examine the relationship between HRR and DR onset and progression in patients with diabetes. We hypothesized that lower HRR is associated with a greater risk of DR onset and progression, independent of traditional risk factors. These findings may provide insights into the mechanisms of DR and support HRR as a potential biomarker for assessing DR risk in diabetic patients.

## 2 Methods

### 2.1 Study population

**Figure 1:**
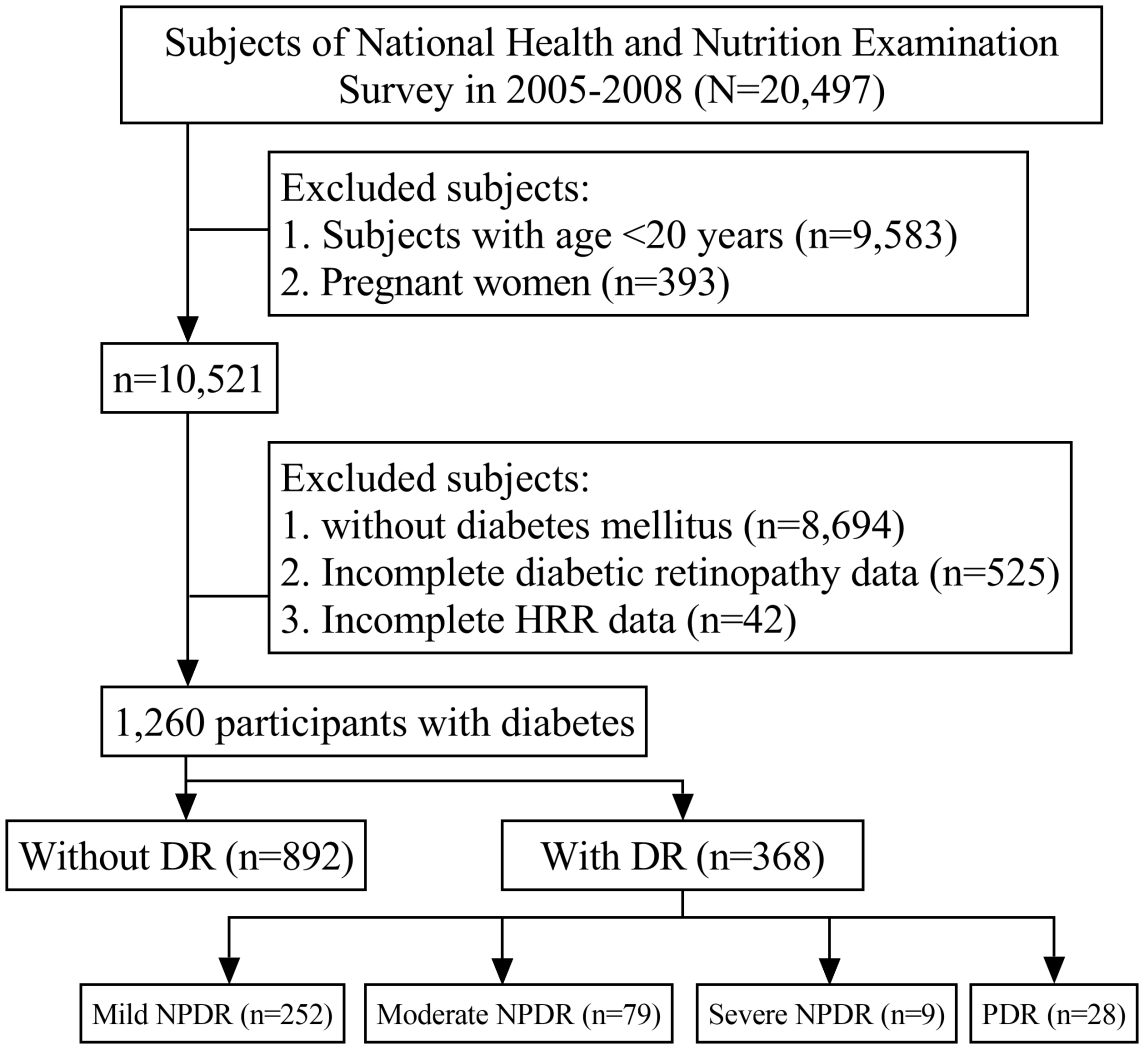
Flowchart of subject selection. The selection of eligible participants in the National Health and Nutrition Examination Survey.

Data were obtained from the 2005–2008 NHANES cycles, a comprehensive health survey conducted by the Centers for Disease Control and Prevention. These cycles were selected for their objective, instrument-based DR diagnoses with detailed severity grading, whereas other cycles rely on self-reported DR diagnoses without severity grading. Participants aged ≥20 years with complete HRR and DR assessment data were included, yielding 1,260 eligible individuals for analysis (**Figure 1**). The National Center for Health Statistics Research Ethics Review Board approved the protocol, and all participants provided written informed consent before enrollment.

### 2.2 Assessment of diabetes and DR

Diabetes was diagnosed using American Diabetes Association criteria (11), supplemented by self-reported data. Participants were classified as diabetic if they met any of the following: Hemoglobin A1c (HbA1c) ≥ 6.5%, fasting plasma glucose ≥ 7 mmol/L, 2-hour plasma glucose ≥ 11.1 mmol/L (oral glucose tolerance test), a physician diagnosis, or use of insulin or other diabetes medications.

DR was evaluated using fundus photography, with retinal features (e.g., microaneurysms, hemorrhages, neovascularization) graded according to the Early Treatment DR Study severity scale (1). Trained NHANES personnel performed 45-degree non-mydriatic fundus photography (CR6-45NM; Canon, Japan) to assess the severity of DR in the worse eye. Experts at the University of Wisconsin graded images according to the NHANES Digital Grading Protocol, classifying DR into five categories: no DR, mild non-proliferative DR (NPDR), moderate NPDR, severe NPDR, or proliferative DR (PDR).

### 2.3 Assessment of hemoglobin-to-red cell distribution width ratio

HRR was calculated from NHANES laboratory data as hemoglobin concentration (g/L) divided by RDW (%). Blood specimens were collected at NHANES Mobile Examination Centers according to standardized protocols. Complete blood count analysis used the Beckman Coulter MAXM analyzer, which employs electrical impedance for cell counting and sizing and a single-beam photometer for hemoglobin measurement. Hemoglobin was measured via cyanmethemoglobin spectrophotometry at 525 nm, and RDW was derived from the red blood cell histogram as the coefficient of variation of the size distribution.

### 2.4 Assessment of covariates

Based on prior DR risk factor studies (1, 2), we adjusted for multiple covariates to control for confounding. These included demographic characteristics (age, sex, race, education level, marital status, poverty income ratio [PIR]), anthropometric measurements (body mass index [BMI], waist circumference), clinical variables (systolic blood pressure [SBP], diastolic blood pressure [DBP], HbA1c, diabetes duration), lipid profile (triglycerides, total cholesterol, high-density lipoprotein cholesterol [HDL-C], low-density lipoprotein cholesterol), renal function markers (serum albumin, serum uric acid, urinary albumin-to-creatinine ratio [UACR], renal failure), lifestyle factors (alcohol consumption, physical activity, smoking), and nutritional and biological indicators (C-reactive protein, vitamin D, energy intake, serum iron, red blood cell count, mean corpuscular volume, white blood cell count, neutrophil percent). Missing data and its handling methods for each covariate are detailed (**Supplementary Table S1**).

### 2.5 Statistical analysis

The NHANES’s complex survey design was accounted for using stratification, sampling weights, and primary sampling units according to analytical guidelines. Descriptive statistics included frequencies and proportions for categorical variables and means ± standard deviations for continuous variables, with group comparisons conducted via weighted linear regression, Wilcoxon rank-sum tests, or chi-square tests as appropriate. The association of HRR with DR (and its subcategories) was evaluated using logistic regression models adjusted for covariates. Restricted cubic splines were used to explore potential non-linear relationships between HRR and DR risk. Mediating effects were evaluated through survey-weighted regression models, with confidence intervals estimated using Monte Carlo simulations. To address confounding, two 1:1 propensity score matching (PSM) analyses were conducted. PSM-1 was based on age, sex, and race. PSM-2 was based on education level, marital status, BMI, alcohol intake, HDL-C, C-reactive protein, vitamin D, energy intake, and physical activity. All analyses were performed in R (version 4.3.1), with a two-sided P value < 0.05 indicating statistical significance.

## 3 Results

### 3.1 Baseline characteristics by DR status

The study included 1,260 diabetic participants from NHANES 2005–2008, of whom 368 had DR and 892 were DR-free (**Table 1**). Demographically, those with DR included a higher proportion of non-Hispanic Black individuals (20.9% vs. 12.9%), a lower PIR (2.64 ± 1.5 vs. 2.93 ± 1.6), and less smoking (46.1% vs. 54.9%) than those without DR. Clinically, they exhibited higher HbA1c (7.7 ± 1.7% vs. 6.7 ± 1.4%), UACR (254 ± 1,054 vs. 65 ± 348 mg/g), longer diabetes duration ≥ 10 years (74.8% vs. 52.1%), and greater renal failure prevalence (9.3% vs. 4.9%), but lower DBP (66.7 ± 14.6 vs. 71.1 ± 14.0 mmHg), serum albumin (4.1 ± 0.4 vs. 4.2 ± 0.3 g/dL), and HRR (10.5 ± 1.7 vs. 11.0 ± 1.7). No differences were observed in age, sex, education, marital status, SBP, BMI, waist circumference, alcohol consumption, lipid profiles, serum uric acid, C-reactive protein, vitamin D, energy intake, physical activity, serum iron, red blood cell count, mean corpuscular volume, white blood cell count, or neutrophil percent (all P > 0.05).

**Table 1:** Baseline characteristics of participants by diabetic retinopathy status among diabetes, NHANES 2005–2008.

Characteristics	DR-free (N=892)	DR (N=368)	*p*-value^a^
Age, mean ± SD (years)	60.86 ± 11.43	62.62 ± 11.51	0.077
Male, NO. (%)	443 (47.3%)	195 (53.7%)	0.110
Race, NO. (%)			0.007
Non-Hispanic White	413 (70.6%)	134 (63.0%)	
Non-Hispanic Black	214 (12.9%)	122 (20.9%)	
Mexican American	171 (7.3%)	75 (8.9%)	
Other	94 (9.2%)	37 (7.3%)	
Education below high school, NO. (%)	583 (56.5%)	248 (59.8%)	0.400
Married/Partner, NO. (%)	554 (67.7%)	224 (64.4%)	0.400
PIR, mean ± SD	2.93 ± 1.58	2.64 ± 1.49	0.038
SBP, mean ± SD (mmHg)	132.05 ± 19.49	135.17 ± 22.85	0.200
DBP, mean ± SD (mmHg)	71.07 ± 14.00	66.65 ± 14.59	<0.001
BMI, mean ± SD (kg/m^2^)	32.48 ± 7.26	32.12 ± 6.64	0.600
Waist circumference, mean ± SD (cm)	109.71 ± 15.90	108.10 ± 15.52	0.200
HbA1c(%), mean ± SD	6.65 ± 1.35	7.71 ± 1.74	<0.001
Diabetes duration (≥10 years), NO. (%)	476 (52.1%)	273 (74.8%)	<0.001
Alcohol intake, NO. (Yes %)	185 (19.5%)	84 (22.1%)	0.400
Smoking, NO. (Yes %)	501 (54.9%)	178 (46.1%)	0.031
Triglyceride (<150 mg/dL), NO. (%)	258 (30.0%)	127 (36.5%)	0.100
Total cholesterol, mean ± SD (mg/dL)	191.04 ± 46.91	185.99 ± 48.37	0.071
HDL-c, mean ± SD (mg/dL)	48.45 ± 14.81	49.57 ± 13.50	0.066
LDL-c (<100 mg/dL %), NO. (%)	221 (26.9%)	100 (28.6%)	0.600
Serum albumin, mean ± SD (g/dL)	4.15 ± 0.32	4.07 ± 0.36	0.013
serum uric acid, mean ± SD (mg/dL)	5.87 ± 1.53	5.75 ± 1.74	0.120
Serum iron (<10.6 µmol/L), NO. (%)	170 (17.0%)	67 (17.9%)	0.932
Red blood cell count, mean eaSD (million cells/onr	4.70 ± 0.50	4.62 ± 0.54	0.063
Mean corpuscular volume, mean eaSD (fL)	89.02 ± 5.87	88.23 ± 5.93	0.089
White blood cell count, mean eaSD (thousand cells/and	7.67 ± 2.30	7.60 ± 3.00	0.312
Neutrophils percent(%), mean eaSD	59.30 ± 9.56	58.99 ± 10.15	0.921
UACR, mean ± SD (mg/g)	64.86 ± 348.45	254.31 ± 1,053.94	<0.001
Renal failure, NO. (Yes %)	51 (4.9%)	36 (9.3%)	0.018
C-reactive protein, mean ± SD (mg/L)	6.79 ± 11.18	5.62 ± 8.81	0.200
Vitamin D (<50 nmol/L), NO. (%)	374 (36.5%)	176 (41.0%)	0.068
Energy intake, mean ± SD (kcal)	1,907.4 ± 823.5	1,854.7 ± 815.6	0.300
MVPA, NO. (Yes %)	432 (51.2%)	158 (49.3%)	0.600
HRR, mean ± SD	10.97 ± 1.69	10.53 ± 1.73	0.002

NHANES, National Health and Nutrition Examination Surveys; DR, diabetic retinopathy; N/NO, sample size; PIR: poverty income ratio; SBP, systolic blood pressure; DBP, diastolic blood pressure; BMI, body mass index; HbA1c, hemoglobin A1c; HDL-c, high-density lipoprotein cholesterol; LDL-c, low-density lipoprotein cholesterol; UACR, urinary albumin/creatinine ratio; MVPA, moderate-vigorous physical activity; HRR, hemoglobin-to-red cell distribution width ratio. Data are represented as mean ± standard deviation or unweighted-n (%). ^a^ P value were calculated using weighted linear regression analyses or wilcoxon rank sum test for continuous variables and the weighted chi-square test for categorical variables.

DR was further categorized into mild NPDR (n = 252), moderate NPDR (n = 79), severe NPDR (n = 9), and PDR (n = 28) (**Supplementary Table S2**). With increasing DR severity, HbA1c, diabetes duration, UACR, proportion of non-Hispanic Black individuals, and renal failure prevalence increased, while PIR and DBP decreased (all P < 0.05). Notably, HRR decreased with increasing DR severity (DR-free: 11.0 ± 1.7; PDR: 9.4 ± 1.6, P = 0.001).

### 3.2 Baseline characteristics of participants by HRR quartiles

Participants were stratified by HRR quartiles, revealing trends across multiple variables (**Supplementary Table S3**). As HRR increased from < 9.62 to > 11.86, age, female proportion, non-Hispanic Black proportion, low education, BMI, HDL-C, UACR, renal failure, C-reactive protein, vitamin D deficiency, and alcohol intake decreased (all P < 0.05). Conversely, male proportion, PIR, DBP, serum albumin, energy intake, and physical activity increased. Notably, DR prevalence decreased across HRR quartiles (36.7%, 23.9%, 24.9%, 19.9%; P = 0.002).

### 3.3 Associations between HRR and DR risk

No multicollinearity was detected among the included variables, with variance inflation factors < 10 and tolerance > 0.1 (**Supplementary Table S4**). Lower HRR was inversely associated with DR risk across all models (**Table 2**), with the fully adjusted model (Model 3; OR = 0.85, β = -0.163, P = 0.008) showing a significant association. HbA1c (OR = 1.58, β = 0.458, P < 0.001) and diabetes duration (≥10 years vs. <10 years: OR = 3.33, β = 1.202, P < 0.001) were also significantly associated with DR risk (**Supplementary Table S5**, Model 3). Stratified analyses (Model 3) showed inverse HRR-DR associations in demographic subgroups, including older adults (≥ 65 years, OR = 0.80, P = 0.009), non-Hispanic White individuals (OR = 0.78, P = 0.008), higher-income individuals (PIR ≥ 2, OR = 0.74, P = 0.004), early-onset diabetes (<30 years, OR = 0.80, P = 0.038), later-onset diabetes (≥30 years, OR = 0.82, P = 0.046), non-insulin users (OR = 0.81, P = 0.040), and insulin users (OR = 0.78, P = 0.048), and clinical subgroups, such as those with normal DBP (< 90 mmHg, OR = 0.84, P = 0.01), better glycemic control (HbA1c < 7%, OR = 0.82, P = 0.012), longer diabetes duration (≥ 10 years, OR = 0.81, P = 0.007), normal albumin levels (≥ 3.5 g/dL, OR = 0.80, P < 0.001), normal UACR (< 30 mg/g, OR = 0.77, P < 0.001), and no renal failure (OR = 0.86, P = 0.017). PSM-1 confirmed these findings (**Supplementary Table S6**, OR = 0.81, P = 0.003). PSM-2 further confirmed HRR’s association with DR risk, addressing additional confounding (**Supplementary Table S7**, OR = 0.83, P = 0.009).

**Table 2:** Associations between hemoglobin-to-red cell distribution width ratio and risk of diabetic retinopathy among diabetes.

Variable	Model 1 OR (95% CI), *P*	Model 2 OR (95% CI), *P*	Model 3 OR (95% CI), *P*
Hemoglobin-to-red cell distribution width ratio	0.86 (0.78-0.94) 0.001	0.85 (0.77-0.95) 0.002	0.85(0.76-0.96) 0.008
Stratified by age			
20–44 years	0.73 (0.47-1.12) 0.154	0.85 (0.53-1.36) 0.492	0.73 (0.40-1.35) 0.321
45–64 years	0.93 (0.83-1.05) 0.266	0.92 (0.79-1.06) 0.241	0.93 (0.77-1.12) 0.438
≥65 years	0.80 (0.70-0.91) <0.001	0.80 (0.69-0.92) 0.003	0.80 (0.68-0.95) 0.009
Stratified by gender			
male	0.83 (0.73-0.95) 0.005	0.83 (0.72-0.96) 0.012	0.83 (0.70-0.99) 0.043
female	0.81 (0.70-0.93) 0.003	0.85 (0.72-0.99) 0.035	0.84 (0.71-0.99) 0.039
Stratified by race			
Non-Hispanic White	0.85 (0.74-0.97) 0.017	0.82 (0.71-0.95) 0.007	0.78 (0.66-0.94) 0.008
Non-Hispanic Black	0.92 (0.80-1.07) 0.282	0.92 (0.79-1.07) 0.279	0.96 (0.80-1.14) 0.633
Mexican American	1.08 (0.91-1.28) 0.384	1.03 (0.84-1.26) 0.772	0.98 (0.77-1.26) 0.893
Other	0.82 (0.57-1.19) 0.305	0.79 (0.51-1.22) 0.284	0.97 (0.63-1.49) 0.896
Stratified by PIR			
<2	0.93 (0.83-1.04) 0.198	0.93 (0.82-1.06) 0.289	0.93 (0.80-1.08) 0.347
≥2	0.79 (0.69-0.92) 0.002	0.75 (0.63-0.89) 0.001	0.74 (0.60-0.91) 0.004
Stratified by DBP			
<90 mmHg	0.87 (0.79-0.96) 0.005	0.86 (0.77-0.96) 0.007	0.84 (0.74-0.96) 0.010
≥90 mmHg	0.96 (0.73-1.28) 0.799	1.07 (0.75-1.54) 0.704	1.20 (0.76-1.90) 0.429
Stratified by HbA1c(%)			
<7	0.79 (0.71-0.89) <0.001	0.78 (0.69-0.89) <0.001	0.82 (0.70-0.96) 0.012
≥7	0.87 (0.76-1.00) 0.050	0.86 (0.73-1.02) 0.085	0.87 (0.72-1.05) 0.153
Stratified by age of diabetes onset			
<30 years	0.82 (0.70tesedic 0.032	0.81 (0.69tesedic 0.035	0.80 (0.68tesedic 0.038
≥30 years	0.84 (0.73tesedic 0.041	0.83 (0.72tesedic 0.043	0.82 (0.70tesedic 0.046
Stratified by Insulin usage			
no	0.83 (0.72iniedic 0.034	0.82 (0.71iniedic 0.037	0.81 (0.69iniedic 0.039
yes	0.80 (0.68iniedic 0.042	0.79 (0.67iniedic 0.045	0.78 (0.66iniedic 0.047
Stratified by diabetes duration			
<10 years	1.03 (0.86-1.22) 0.779	1.01 (0.84-1.21) 0.942	0.96 (0.77-1.20) 0.733
≥10 years	0.82 (0.73-0.92) <0.001	0.82 (0.72-0.93) 0.002	0.81 (0.69-0.94) 0.007
Stratified by Smoking			
no	0.89 (0.77-1.02) 0.087	0.84 (0.71-0.99) 0.038	0.83 (0.69-1.00) 0.048
yes	0.84 (0.76-0.94) 0.003	0.86 (0.76-0.97) 0.019	0.88 (0.75-0.98) 0.028
Stratified by Serum albumin			
<3.5 g/dL	1.58 (0.97-2.57) 0.076	2.09 (1.12-3.92) 0.028	2.59 (1.02-6.62) 0.058
≥3.5 g/dL	0.84 (0.77-0.93) <0.001	0.82 (0.73-0.91) <0.001	0.80 (0.71-0.91) <0.001
Stratified by UACR			
<30 mg/g	0.83 (0.73-0.93) 0.002	0.78 (0.68-0.90) <0.001	0.77 (0.66-0.90) <0.001
30–300 mg/g	1.00 (0.85-1.16) 0.951	1.10 (0.92-1.30) 0.298	1.15 (0.94-1.42) 0.175
>300 mg/g	1.07 (0.82-1.40) 0.630	1.17 (0.86-1.59) 0.333	1.18 (0.75-1.85) 0.471
Stratified by renal failure			
no	0.87 (0.80-0.96) 0.005	0.86 (0.77-0.96) 0.009	0.86 (0.76-0.97) 0.017
yes	0.86 (0.62-1.19) 0.360	0.86 (0.58-1.26) 0.430	0.87 (0.58-1.30) 0.498

OR, odds ratio; 95% CI, 95% confidence interval; PIR: poverty income ratio; DBP, diastolic blood pressure; HbA1c, hemoglobin A1c; UACR, urinary albumin/creatinine ratio.

Mode 1 = Non-adjusted mode.

Mode 2 = Mode 1 + age, gender, and race were adjusted.

Model 3 = Mode 2 + PIR, DBP, HbA1c(%), diabetes duration, smoking, serum albumin, UACR and renal failure. The subgroup analysis was not adjusted for the stratification variable itself.

**Figure 2:**
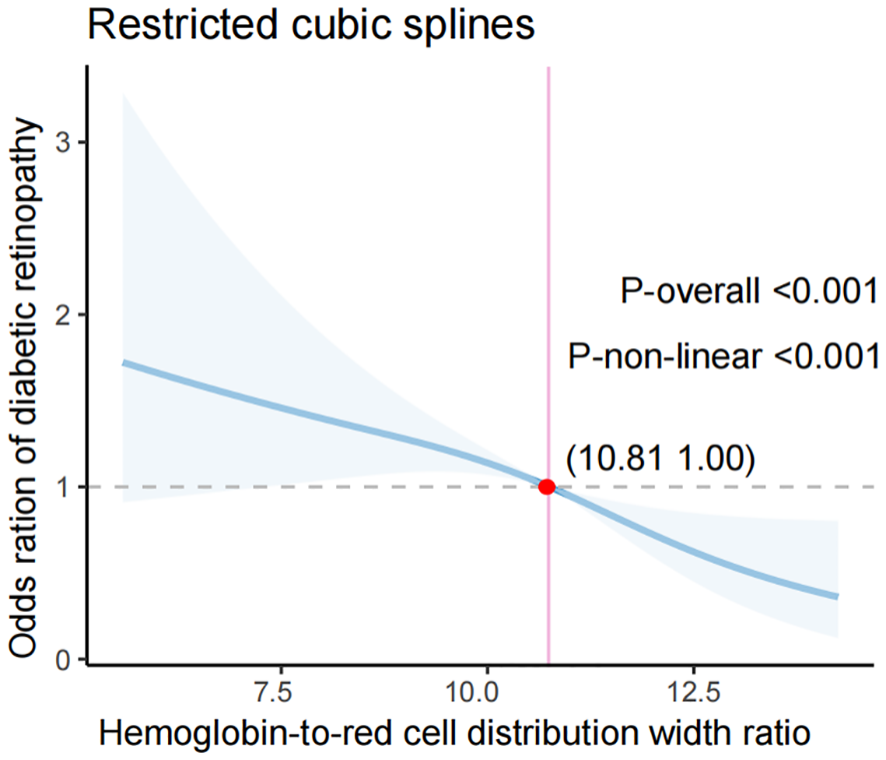
Non-linear association between hemoglobin-to-red cell distribution width ratio and diabetic retinopathy using restricted cubic splines.

Restricted cubic spline analysis revealed a non-linear HRR-DR relationship with an inflection point at HRR = 10.81 (P for non-linearity < 0.001) (**Figure 2**). Below HRR = 10.81, this relationship was positive but weakened with increasing HRR; above HRR = 10.81, it was negative and strengthened with increasing HRR.

### 3.4 Mediating variables in the relationship between HRR and DR risk

**Figure 3:**
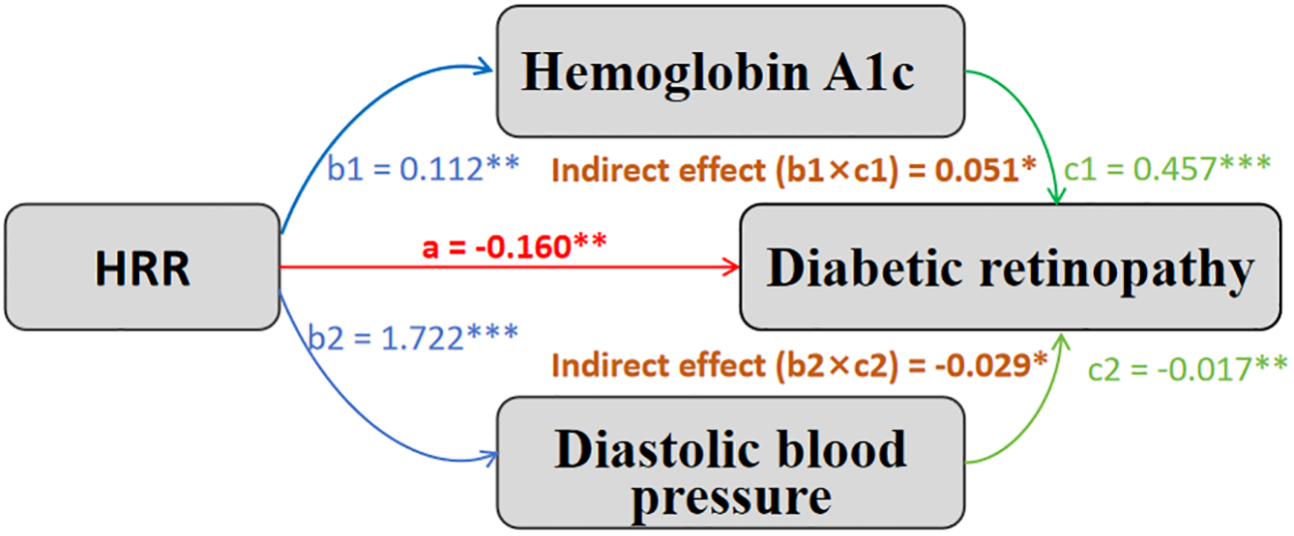
Mediating pathways in the association between HRR and diabetic retinopathy. HRR, Hemoglobin-to-red cell distribution width ratio. — a path: Direct effect of HRR on diabetic retinopathy. — b path: Effect of HRR on mediator variable. — c path: Effect of mediator variable on diabetic retinopathy. *p < 0.05, **p < 0.01, ***p < 0.001.

DBP and HbA1c were identified as mediators of the HRR-DR relationship. DBP mediated 15.9% of the HRR-DR effect (coefficient: -0.029; 95% CI: -0.056, -0.008; P = 0.013) (**Figure 3**). Path analysis showed HRR positively associated with DBP (coefficient = 1.722, P < 0.001), which was negatively associated with DR risk (coefficient = -0.017, P = 0.006), with a significant direct effect (coefficient = -0.160, P = 0.008), confirming partial mediation. In contrast, HbA1c showed competitive mediation, counteracting 60.5% of the HRR-DR effect (**Figure 3**). HRR was positively associated with HbA1c (coefficient = 0.112, P = 0.009), which increased DR risk (coefficient = 0.457, P < 0.001), partially offsetting HRR’s protective effect (indirect effect: coefficient = 0.051, P = 0.014). Other variables (PIR, serum albumin, UACR) showed no significant mediation (**Supplementary Table S8**).

### 3.5 Association between HRR and DR severity

We assessed HRR associations with DR severity across severity pairings (**Table 3**). Compared to mild NPDR, higher HRR reduced PDR odds (OR = 0.67, 95% CI: 0.47–0.96, P = 0.030); compared to severe NPDR, HRR also reduced PDR odds (OR = 0.04, 95% CI: 0.01–0.08, P = 0.002). No significant HRR associations were found for mild vs. moderate NPDR (P = 0.96), mild vs. severe NPDR (P = 0.401), moderate vs. severe NPDR (P = 0.140), or moderate NPDR vs. PDR (P = 0.065).

**Table 3:** Impact of hemoglobin-to-red cell distribution width ratio on DR severity.

DR status pairings	Model 1 OR (95% CI), *P*	Model 2 OR (95% CI), *P*	Model 3 OR (95% CI), *P*
Mild NPDR and Moderate NPDR	0.98 (0.83-1.16) 0.801	1.00 (0.83-1.20) 0.972	0.99 (0.79-1.25) 0.960
Mild NPDR and Severe NPDR	1.11 (0.75-1.63) 0.616	1.43 (0.90-2.27) 0.132	1.35 (0.67-2.68) 0.401
Mild NPDR and PDR	0.69 (0.52-0.91) 0.010	0.69 (0.48-0.97) 0.034	0.67 (0.47-0.96) 0.030
Moderate NPDR and Severe NPDR	1.17 (0.68-2.03) 0.566	2.36 (1.08-5.16) 0.035	6.35 (0.56-72.1) 0.140
Moderate NPDR and PDR	0.64 (0.41-0.98) 0.040	0.57 (0.32-1.02) 0.060	0.48 (0.22-1.04) 0.065
Severe NPDR and PDR	0.55 (0.27-1.10) 0.098	0.11 (0.02-0.61) 0.017	0.04 (0.01-0.08) 0.002

OR, odds ratio; 95% CI, 95% confidence interval; DR, diabetic retinopathy; NPDR: non-proliferative diabetic retinopathy; PDR: proliferative diabetic retinopathy.

Mode 1 = Non-adjusted mode.

Mode 2 = Mode 1 + age, gender, and race were adjusted.

Model 3 = Mode 2 + poverty income ratio, diastolic blood pressure, hemoglobin A1c(%), diabetes duration, smoking, serum albumin, UACR and renal failure.

For each paired analysis, the reference category is the non-diseased or milder condition.

## 4 Discussion

This study investigated the association of the HRR with DR risk and severity in patients with diabetes. Our findings show a significant inverse relationship between HRR and both DR development and severity, consistent after PSM-1 and PSM-2. This suggests that HRR could serve as a simple, cost-effective biomarker to identify diabetic patients at higher risk of DR.

This protective effect of higher HRR is consistent with its established prognostic value across multiple disease states. In cardiovascular medicine, Sun et al. reported reduced cardiovascular mortality in patients with diabetes with increasing HRR quartiles (hazard ratios: 0.48, 0.44, 0.39 for ascending quartiles vs. lowest) (6). Xiu et al. found that an HRR < 10.25 was associated with increased mortality following percutaneous coronary intervention (adjusted hazard ratio: 1.479) (12). In stroke research, Wang et al. reported that each unit increase in HRR reduced the likelihood of unfavorable outcomes by 10% in patients with acute ischemic stroke (5). Qin et al. noted that for HRR values ≤ 9.74, each unit increase was associated with a 27% decrease in all-cause mortality in patients with ischemic stroke with atrial fibrillation (13). Gao et al. observed that a low HRR at 24 hours was associated with a poor prognosis (adjusted odds ratio: 0.646) and increased mortality (adjusted odds ratio: 0.615) in patients undergoing thrombectomy (10). In oncology, Chi et al.’s meta-analysis showed that a low HRR doubled the risk of all-cause mortality (hazard ratio: 2.29) and disease progression (hazard ratio: 2.19) (9). Liu et al. reported that a lower HRR was associated with an increased likelihood of breast cancer diagnosis (14). Our study extends this protective association of HRR to DR, providing novel evidence for its role in retinal microvascular disease.

Our analysis showed that higher HRR reduced the risk of PDR compared to mild and severe NPDR, but showed no significant associations between earlier NPDR stages. The progression of DR is marked by escalating retinal inflammation and hypoxia, with PDR characterized by extensive neovascularization driven by oxygen deficiency (2). The protective effect of HRR in transitions to PDR highlights its potential as a biomarker for identifying patients at risk of severe DR (1). The small sample size of the severe NPDR group may have limited our ability to detect associations at earlier DR stages, but the strong effect observed for severe NPDR to PDR underscores HRR’s clinical utility for identifying proliferative complications.

The protective role of higher HRR in DR pathogenesis likely involves several biological mechanisms. First, a higher HRR reflects lower systemic inflammation, a critical factor in DR (2). Inflammatory cytokines disrupt erythropoiesis by altering iron metabolism and promoting the release of immature erythrocytes, thereby increasing RDW and reducing HRR (14). These cytokines also impair retinal vascular endothelial function, leading to microcirculatory dysfunction and exacerbating DR progression (2). Second, a lower HRR signals impaired oxygen delivery due to reduced hemoglobin levels and increased red cell heterogeneity (higher RDW). Lower hemoglobin reduces oxygen delivery to retinal tissues, while a higher RDW is associated with reduced erythrocyte deformability, which impedes microcirculation and contributes to retinal capillary non-perfusion (10). Such microcirculatory impairment may amplify hypoxia, thereby upregulating vascular endothelial growth factor expression and promoting pathological angiogenesis in DR (6). Third, the higher prevalence of anemia among patients with diabetes may accelerate DR onset via mechanisms induced by tissue hypoxia (6). By integrating hemoglobin and RDW, HRR offers a more comprehensive evaluation of inflammation, oxygen-carrying capacity, and microcirculatory function than either component alone, thereby enhancing its predictive value for DR (15). Collectively, these mechanisms underlie the protective effect of higher HRR on DR onset and progression.

Our analysis revealed a significant non-linear relationship between HRR and DR risk, characterized by an inflection point at HRR = 10.81. Below this threshold, lower HRR values were associated with an increased DR risk, whereas values above 10.81 conferred greater protection. This non-linear pattern suggests a physiological threshold at which HRR’s effects on inflammation and oxygen delivery become more pronounced, a finding consistent with similar inflection points reported in other conditions. For instance, Wang et al. identified non-linear associations between HRR and acute ischemic stroke with inflection points at 10.57 and 10.70 (5, 16), and Qin et al. reported an inflection point at 9.74 for mortality in ischemic stroke patients with atrial fibrillation (13). The consistency of these inflection points (9.74–10.81) across diverse disease states highlights HRR’s utility as a robust biomarker with a critical threshold for predicting outcomes.

Subgroup analyses revealed that the protective effect of higher HRR in reducing DR risk was significant in specific groups. This significant effect was observed in patients aged ≥ 65 years and those with longer diabetes duration (≥ 10 years) and may reflect the lower baseline HRR values in these groups, which makes the protective effects more apparent. The observed racial and socioeconomic differences in the protective effects of HRR may result from variations in nutritional status, healthcare access, and adherence to treatment, all of which can affect HRR levels and DR risk. Among patients with normal clinical parameters (such as blood pressure, glycemic control, and renal function), the protective effect of higher HRR was evident, possibly because these patients typically exhibit better vascular function and lower inflammatory status (8), which may enable more efficient use of the oxygen delivered by the high oxygen-carrying capacity associated with higher HRR (17). This is supported by our baseline data, which show significantly lower C-reactive protein levels among those with higher HRR, suggesting a mechanistic link among HRR, levels of inflammation, and retinal microvascular health.

Our mediation analysis revealed two contrasting mechanisms in the relationship between HRR and DR. DBP mediated 15.9% of the protective effect of HRR. The positive association of HRR with DBP suggests that a higher HRR may enhance vascular function through improved oxygen-carrying capacity and reduced inflammation (10), while a higher DBP (within the normal range) may reduce the risk of DR by supporting retinal blood flow (16). Our baseline data indicate a lower DBP among those with DR compared to those without, although both groups’ values were within the normal range. In contrast, HbA1c exhibited competitive mediation, offsetting 60.5% of the protective effect of HRR. The positive relation of HRR with HbA1c likely arises from two complementary mechanisms: higher hemoglobin levels provide more material for glycation, and lower RDW may reflect reduced red blood cell turnover, both promoting the accumulation of glycated hemoglobin (18). Despite this counteracting effect, the direct protective effect of HRR outweighs this negative influence, resulting in an overall protective effect against DR. However, these mediation findings should be interpreted cautiously due to the cross-sectional design’s limitation in establishing causal directionality. In this study, established risk factors like HbA1c and diabetes duration exhibit larger regression coefficients, indicating stronger contributions to increased DR risk compared to the protective effect of HRR, which remains statistically significant but relatively modest.

This study leverages a large, nationally representative NHANES sample, enhancing its generalizability, and incorporates comprehensive clinical and biochemical data with rigorous statistical methods to evaluate a novel, cost-effective biomarker. Stratified analyses by age of diabetes onset and insulin use as proxy indicators for diabetes types consistently demonstrated negative associations between HRR and DR risk across subgroups.

Limitations of this study include its cross-sectional design, which precludes causal inference and limits confirmation of mediation effect directionality due to potential reverse causality, reliance on single-time HRR measurements, potential recall bias from self-reported data, and the inability to definitively distinguish diabetes types. Additionally, covariates not considered in this study or not significantly associated with DR in the baseline characteristics table may still introduce minor residual confounding. Furthermore, the NHANES 2005–2008 cycles lack data on glycated albumin (GA) or other glycemic variability markers, such as 1,5-anhydroglucitol or fructosamine, which could have provided a more comprehensive assessment of DR risk. GA, reflecting 2–3 weeks of glycemic fluctuations and more sensitive than HbA1c to short-term variability, is elevated in DR patients (19). Lower ratio of glucose management index to GA are associated with higher DR risk in patients with diabetes (20). As the NHANES sample is derived from a U.S. population, the findings may have limited generalizability, necessitating external validation in international and ethnically diverse cohorts to confirm HRR’s predictive utility for DR risk.

Future research should prioritize prospective cohort studies in international and ethnically diverse populations to validate HRR’s predictive value and generalizability, establish causality by tracking HRR and DR progression over time, explore its relationship with other diabetic complications, and evaluate interventions targeting HRR to reduce DR risk. Future DR studies should ideally incorporate GA alongside other markers to enhance risk prediction. These studies should employ precise diabetes type classification to refine subgroup analyses, incorporate repeated HRR measurements to assess intra-individual variability, and include larger sample sizes to enhance statistical power for early DR stage analyses. Additionally, integrating robust biomarkers of iron metabolism (e.g., transferrin saturation, total iron binding capacity) and erythropoiesis (e.g., erythropoietin, reticulocyte count) will better control for these pathways. Molecular studies may further clarify the pathophysiological mechanisms of HRR, potentially revealing novel therapeutic targets for DR management.

## 5 Conclusion

This study demonstrates a significant inverse relationship between HRR and both DR onset and severity in patients with diabetes. These results suggest that HRR could serve as a novel, cost-effective biomarker for assessing the risk of DR in patients with diabetes.

## Data Availability

The original contributions presented in the study are included in the article/**Supplementary Material**. Further inquiries can be directed to the corresponding author.
