# Supplementary material for: Association of hemoglobin-to-red cell distribution width ratio with diabetic retinopathy risk and severity
Source: Front Endocrinol (Lausanne). 2025 Aug 11;16:1622460. doi: 10.3389/fendo.2025.1622460 (PMC12375449; doi:10.3389/fendo.2025.1622460)
Supplement: Supplementary file 1 [file Table1.docx]

**Association of hemoglobin-to-red cell distribution width ratio with diabetic retinopathy risk and severity**

Bin Wang, Hui Li, Lin Wang, Zaihong Chen

**Online supplementary material**

**Table S1.** Missingness and treatment of each covariate

**Table S2.** Baseline characteristics of participants by severity of diabetic retinopathy among diabetes patients

**Table S3.** Baseline characteristics of participants by HRR among participants

**Table S4.** Collinearity statistics of included variables

**Table S5.** Associations between included varaibles and risk of diabetic retinopathy in fully adjusted model

**Table S6.** Associations between hemoglobin-to-red cell distribution width ratio and risk of diabetic retinopathy among diabetes after PSM-1

**Table S7.** Associations between hemoglobin-to-red cell distribution width ratio and risk of diabetic retinopathy among diabetes after PSM-2

**Table S8.** Potential mediating variables of hemoglobin-to-red cell distribution width ratio on diabetic retinopathy risk

| **Table S1.** Missingness and treatment of each covariate | | |
| --- | --- | --- |
| Variables | Missing quantity | Processing of missing data |
| Age | 0 | - |
| Gender | 0 | - |
| Race | 0 | - |
| Education level | 0 | - |
| Marital status | 0 | - |
| Poverty income ratio | 30 | a |
| Body mass index | 14 | a |
| Waist circumference | 38 | a |
| Systolic blood pressure | 85 | a |
| Diastolic blood pressure | 85 | a |
| Hemoglobin A1c | 6 | a |
| Diabetes duration | 0 | - |
| Smoking | 2 | - |
| Alcohol intake | 769 | b |
| Triglyceride | 546 | b |
| Total cholesterol | 10 | a |
| High-density lipoprotein cholesterol | 10 | a |
| Low-density lipoprotein cholesterol | 586 | b |
| Serum albumin | 20 | a |
| Serum uric acid | 21 | a |
| Serum iron | 427 | b |
| Red blood cell count | 0 | - |
| Mean corpuscular volume | 0 | - |
| White blood cell count | 1 | a |
| Neutrophils percent | 7 | a |
| Urinary albumin/creatinine ratio | 20 | a |
| Renal failure | 2 | b |
| C-reactive protein | 5 | a |
| Vitamin D | 84 | b |
| Energy intake | 25 | a |
| Moderate to vigorous physical activity | 0 | - |
| a, Fill in missing values with median; b, Using missing values as a categorical variable | | |

| **Table S2**. Baseline characteristics of participants by severity of diabetic retinopathy among diabetes patients | | | | | | |
| --- | --- | --- | --- | --- | --- | --- |
| Characteristics | DR-free  (N=892) | Mild NPDR  (N=252) | Moderate NPDR (N=79) | Severe NPDR  (N=9) | PDR  (N=28) | *p*-value^a^ |
| Age, mean±SD (years) | 60.86 ± 11.43 | 63.38 ± 11.73 | 59.48 ± 11.06 | 66.04 ± 12.29 | 62.85 ± 8.32 | 0.083 |
| Male, NO. (%) | 443 (47.3%) | 141 (55.3%) | 40 (49.3%) | 3 (38.6%) | 11 (54.8%) | 0.400 |
| Race, NO. (%) |  |  |  |  |  | 0.004 |
| Non-Hispanic White | 413 (70.6%) | 106 (67.0%) | 21 (54.6%) | 2 (41.3%) | 5 (50.1%) |  |
| Non-Hispanic Black | 214 (12.9%) | 71 (17.1%) | 34 (27.9%) | 4 (41.7%) | 13 (35.3%) |  |
| Mexican American | 171 (7.3%) | 47 (7.5%) | 20 (13.1%) | 3 (17.0%) | 5 (7.6%) |  |
| Other | 94 (9.2%) | 28 (8.4%) | 4 (4.3%) | 0 (0.0%) | 5 (7.0%) |  |
| Education below high school, NO.(%) | 583 (56.5%) | 170 (60.3%) | 54 (57.0%) | 5 (76.4%) | 19 (58.1%) | 0.800 |
| Married/Partner, NO. (%) | 554 (67.7%) | 156 (65.2%) | 51 (67.6%) | 4 (23.6%) | 13 (57.8%) | 0.200 |
| PIR, mean±SD | 2.93 ± 1.58 | 2.60 ± 1.45 | 3.10 ± 1.62 | 2.21 ± 1.72 | 1.76 ± 0.89 | <0.001 |
| SBP, mean±SD (mmHg) | 132.05 ± 19.49 | 136.14 ± 23.47 | 130.34 ± 21.17 | 141.84 ± 19.31 | 137.79 ± 20.34 | 0.120 |
| DBP, mean±SD (mmHg) | 71.07 ± 14.00 | 66.56 ± 15.75 | 67.37 ± 10.55 | 68.59 ± 13.91 | 64.58 ± 12.17 | <0.001 |
| BMI, mean±SD (kg/m^2^) | 32.48 ± 7.26 | 31.45 ± 6.05 | 34.54 ± 7.85 | 32.07 ± 6.32 | 31.97 ± 7.56 | 0.200 |
| Waist circumference, mean±SD (cm) | 109.71 ± 15.90 | 106.27 ± 14.23 | 113.83 ± 18.72 | 109.57 ± 14.21 | 110.24 ± 14.72 | 0.072 |
| HbA1c(%), mean±SD | 6.65 ± 1.35 | 7.48 ± 1.63 | 8.39 ± 1.90 | 7.99 ± 1.04 | 8.16 ± 2.02 | <0.001 |
| Diabetes duration (≥10 years),NO.(%) | 476 (52.1%) | 174 (69.5%) | 66 (86.0%) | 7 (86.9%) | 26 (97.0%) | <0.001 |
| Alcohol intake, NO. (Yes %) | 185 (19.5%) | 55 (20.7%) | 21 (28.4%) | 1 (11.2%) | 7 (20.7%) | 0.600 |
| Smoking, NO. (Yes %) | 501 (54.9%) | 127 (49.1%) | 35 (34.4%) | 5 (74.8%) | 11 (40.6%) | 0.120 |
| Triglyceride (<150 mg/dL %), NO.(%) | 258 (30.0%) | 92 (38.0%) | 24 (32.2%) | 3 (42.0%) | 8 (30.1%) | 0.500 |
| Total cholesterol, mean±SD (mg/dL) | 191.04 ± 46.91 | 185.33 ± 48.04 | 183.21 ± 51.20 | 222.03 ± 40.49 | 191.44 ± 41.62 | 0.055 |
| HDL-c, mean±SD (mg/dL)) | 48.45 ± 14.81 | 50.04 ± 12.99 | 47.36 ± 14.80 | 53.76 ± 14.20 | 49.95 ± 14.70 | 0.120 |
| LDL-c (<100 mg/dL %), NO. (%) | 221 (26.9%) | 76 (29.7%) | 18 (28.9%) | 2 (22.4%) | 4 (16.4%) | 0.800 |
| Serum albumin, mean±SD (g/dL) | 4.15 ± 0.32 | 4.11 ± 0.35 | 3.95 ± 0.38 | 4.24 ± 0.39 | 4.06 ± 0.28 | 0.002 |
| serum uric acid, mean±SD (mg/dL) | 5.87 ± 1.53 | 5.72 ± 1.76 | 5.71 ± 1.59 | 5.69 ± 1.03 | 6.16 ± 2.16 | 0.500 |
| UACR, mean±SD (mg/g) | 64.86 ± 348.45 | 162.28 ± 724.06 | 372.07 ± 1,023.03 | 512.34 ± 1,426.89 | 891.34 ± 2,834.89 | <0.001 |
| Renal failure, NO. (Yes %) | 51 (4.9%) | 22 (7.1%) | 5 (4.0%) | 0 (0.0%) | 9 (57.2%) | <0.001 |
| C-reactive protein, mean±SD (mg/L) | 6.79 ± 11.18 | 5.10 ± 9.19 | 6.86 ± 7.55 | 4.85 ± 6.80 | 8.04 ± 8.37 | 0.095 |
| Vitamin D, mean±SD (nmol/L) | 374 (36.5%) | 108 (38.1%) | 46 (45.0%) | 5 (46.7%) | 17 (60.5%) | 0.140 |
| Energy intake, mean±SD (kcal) | 1,907.4 ± 823.5 | 1,837.6 ± 775.0 | 2,006.9 ± 978.0 | 1,344.6 ± 706.3 | 1,711.7 ± 622.3 | 0.100 |
| MVPA, NO. (Yes %) | 432 (51.2%) | 117 (50.2%) | 34 (51.1%) | 3 (46.7%) | 4 (32.3%) | 0.800 |
| HRR, mean±SD | 10.97 ± 1.69 | 10.61 ± 1.77 | 10.54 ± 1.58 | 10.91 ± 1.39 | 9.38 ± 1.57 | 0.001 |
| Abbreviations: NHANES, National Health and Nutrition Examination Surveys; DR, diabetic retinopathy; M-NPDR: mild non-proliferative diabetic retinopathy; MS-NPDR: moderate/severe non-proliferative diabetic retinopathy; PDR: proliferative diabetic retinopathy; N/NO, sample size; PIR: poverty income ratio; SBP, systolic blood pressure; DBP, diastolic blood pressure; BMI, body mass index (calculated as weight in kilograms divided by height in square meters); HbA1c, hemoglobin A1c; HDL-c, high-density lipoprotein cholesterol; LDL-c, low-density lipoprotein cholesterol; UACR, urinary albumin/creatinine ratio; MVPA, moderate-vigorous physical activity; HRR, hemoglobin-to-red cell distribution width ratio. Data are represented as mean ± standard deviation or unweighted-n (%). ^a^ P value were calculated using wilcoxon rank sum test for continuous variables and the weighted chi-square test with Rao & Scott's second-order correction for categorical variables. | | | | | | |

| **Table S3.** Baseline characteristics of participants by HRR among participants | | | | | |
| --- | --- | --- | --- | --- | --- |
| Characteristics | <9.62 (N=315) | 9.62-10.75 (N=315) | 10.76-11.86 (N=315) | >11.86 (N=315) | *p*-value^a^ |
| Age, mean±SD (years) | 63.57 ± 11.70 | 62.11 ± 12.15 | 60.37 ± 11.43 | 59.85 ± 10.42 | 0.006 |
| Male, NO. (%) | 103 (30.9%) | 126 (32.8%) | 165 (49.7%) | 244 (75.6%) | <0.001 |
| Race, NO. (%) |  |  |  |  | <0.001 |
| Non-Hispanic White | 547 (68.7%) | 96 (55.0%) | 132 (66.8%) | 147 (73.0%) |  |
| Non-Hispanic Black | 336 (15.0%) | 151 (32.3%) | 100 (17.4%) | 60 (10.8%) |  |
| Mexican American | 246 (7.7%) | 46 (6.9%) | 52 (5.9%) | 67 (7.3%) |  |
| Other | 131 (8.7%) | 22 (5.9%) | 31 (9.8%) | 41 (8.8%) |  |
| Education below high school, NO. (%) | 212 (63.3%) | 217 (63.0%) | 210 (58.5%) | 192 (47.0%) | 0.004 |
| Married/Partner, NO. (%) | 164 (56.4%) | 189 (65.9%) | 189 (66.6%) | 236 (75.5%) | 0.001 |
| PIR, mean±SD | 2.43 ± 1.40 | 2.70 ± 1.48 | 3.04 ± 1.66 | 3.12 ± 1.57 | <0.001 |
| SBP, mean±SD (mmHg) | 133.49 ± 22.16 | 133.58 ± 20.21 | 131.64 ± 20.92 | 132.90 ± 18.84 | 0.800 |
| DBP, mean±SD (mmHg) | 64.70 ± 15.12 | 68.26 ± 16.12 | 70.92 ± 13.20 | 74.30 ± 11.13 | <0.001 |
| BMI, mean±SD (kg/m^2^) | 34.20 ± 8.46 | 31.96 ± 6.98 | 32.20 ± 7.18 | 31.62 ± 5.74 | 0.018 |
| Waist circumference, mean±SD (cm) | 109.90 ± 16.89 | 107.37 ± 16.37 | 109.84 ± 15.99 | 110.01 ± 14.18 | 0.500 |
| HbA1c(%), mean±SD | 6.89 ± 1.48 | 6.73 ± 1.19 | 7.04 ± 1.55 | 7.01 ± 1.79 | 0.400 |
| Diabetes duration (≥10 years), NO. (%) | 203 (65.2%) | 179 (53.7%) | 174 (54.0%) | 193 (59.9%) | 0.500 |
| DR, NO. (Yes %) | 119 (36.7%) | 89 (23.9%) | 84 (24.9%) | 76 (19.9%) | 0.002 |
| Alcohol intake, NO. (Yes %) | 79 (26.1%) | 77 (22.8%) | 57 (17.7%) | 56 (15.8%) | 0.001 |
| Smoking, NO. (Yes %) | 150 (45.7%) | 169 (53.3%) | 168 (53.5%) | 192 (56.4%) | 0.400 |
| Triglyceride (<150 mg/dL %), NO. (%) | 109 (32.5%) | 96 (35.1%) | 94 (28.2%) | 86 (31.2%) | 0.082 |
| Total cholesterol, mean±SD (mg/dL) | 183.77 ± 43.54 | 189.44 ± 46.20 | 187.66 ± 51.12 | 196.33 ± 46.64 | 0.062 |
| HDL-c, mean±SD (mg/dL)) | 51.60 ± 16.26 | 50.47 ± 15.06 | 47.41 ± 13.36 | 46.38 ± 13.08 | 0.002 |
| LDL-c (<100 mg/dL %), NO. (%) | 85 (26.4%) | 81 (29.4%) | 78 (25.8%) | 77 (27.7%) | 0.600 |
| Serum albumin, mean±SD (g/dL) | 3.94 ± 0.37 | 4.12 ± 0.28 | 4.14 ± 0.31 | 4.27 ± 0.29 | <0.001 |
| serum uric acid, mean±SD (mg/dL) | 6.10 ± 2.13 | 5.88 ± 1.49 | 5.66 ± 1.38 | 5.78 ± 1.34 | 0.400 |
| UACR, mean±SD (mg/g) | 225.47 ± 1,015.69 | 97.84 ± 436.15 | 117.79 ± 644.71 | 41.32 ± 175.03 | <0.001 |
| Renal failure, NO. (Yes %) | 46 (14.7%) | 17 (4.8%) | 13 (3.6%) | 11 (3.0%) | <0.001 |
| C-reactive protein, mean±SD (mg/L) | 10.55 ± 16.60 | 6.19 ± 7.17 | 5.93 ± 10.17 | 4.29 ± 6.39 | <0.001 |
| Vitamin D, mean±SD (mg/L) | 171 (48.2%) | 144 (42.3%) | 129 (33.1%) | 106 (30.2%) | <0.001 |
| Energy intake, mean±SD (kcal) | 1,653.51 ± 748.59 | 1,766.12 ± 706.23 | 1,913.81 ± 753.98 | 2,162.90 ± 942.91 | <0.001 |
| MVPA, NO. (Yes %) | 101 (33.2%) | 157 (54.6%) | 158 (54.9%) | 174 (56.1%) | <0.001 |
| Abbreviations: HRR, hemoglobin-to-red cell distribution width ratio; N/NO, sample size; PIR: poverty income ratio; SBP, systolic blood pressure; DBP, diastolic blood pressure; BMI, body mass index (calculated as weight in kilograms divided by height in square meters); HbA1c, hemoglobin A1c; DR, diabetic retinopathy; HDL-c, high-density lipoprotein cholesterol; LDL-c, low-density lipoprotein cholesterol; UACR, urinary albumin/creatinine ratio; MVPA, moderate-vigorous physical activity. Data are represented as mean ± standard deviation or unweighted-n (%). ^a^ P value were calculated using weighted linear regression analyses or wilcoxon rank sum test for continuous variables and the weighted chi-square test for categorical variables. | | | | | |

| **Table S4.** Collinearity statistics of included variables | | |
| --- | --- | --- |
| Variables | VIF | Tolerance |
| Age | 1.23 | 0.80 |
| Gender | 1.23 | 0.81 |
| Race | 1.09 | 0.91 |
| Poverty income ratio | 1.09 | 0.91 |
| Diastolic blood pressure | 1.19 | 0.83 |
| Hemoglobin A1c | 1.09 | 0.91 |
| Diabetes duration | 1.02 | 0.97 |
| Serum albumin | 1.24 | 0.81 |
| Urinary albumin/creatinine ratio | 1.10 | 0.91 |
| Smoking | 1.09 | 0.91 |
| Renal failure | 1.07 | 0.92 |
| Abbreviations: VIF, variance inflation factor. Dependent variable: diabetic retinopathy status. | | |

| Table S5. Associations between included varaibles and risk of diabetic retinopathy in fully adjusted model | | | | |
| --- | --- | --- | --- | --- |
| Variables | OR | β | OR 95% CI | P-Value |
| HRR | 0.85 | -0.162 | 0.76 - 0.96 | 0.008 |
| Age | 1.01 | 0.010 | 0.99 - 1.03 | 0.222 |
| Gender male | reference |  | - | - |
| female | 0.52 | -0.654 | 0.35 - 0.76 | <0.001 |
| Race non-hispanic white | reference |  | - | - |
| non-hispanic black | 1.31 | 0.270 | 0.86 - 1.96 | 0.208 |
| mexican american | 1.01 | 0.010 | 0.62 - 1.63 | 0.977 |
| other | 0.77 | -0.261 | 0.38 - 1.54 | 0.457 |
| poverty income ratio | 0.91 | -0.094 | 0.81 - 1.02 | 0.095 |
| Diastolic blood pressure | 0.98 | -0.020 | 0.97 - 0.99 | 0.006 |
| Hemoglobin A1c(%) | 1.58 | 0.458 | 1.41 - 1.78 | <0.001 |
| Diabetes duration <10 years | reference |  |  |  |
| ≥10 years | 3.33 | 1.202 | 1.95 - 5.71 | <0.001 |
| Smoking no | reference |  |  |  |
| yes | 0.54 | -0.616 | 0.38 - 0.78 | 0.001 |
| Serum albumin | 1.16 | 0.148 | 0.65 - 2.08 | 0.605 |
| Urinary albumin/creatinine ratio | 1.004 | 0.004 | 1.001 - 1.007 | 0.016 |
| Renal failure no | reference |  | - | - |
| yes | 1.27 | 0.239 | 0.66 - 2.45 | 0.466 |
| Abbreviations: OR, odds ratio; β, regression coefficient; 95% CI, 95% confidence interval; HRR, hemoglobin-to-red cell distribution width ratio. HRR, age, gender, race, poverty income ratio, diastolic blood pressure, hemoglobin A1c, diabetes duration, smoking, serum albumin, urinary albumin/creatinine ratio and renal failure were included in the weighted logistic regression model. | | | | |

| **Table S6.** Associations between hemoglobin-to-red cell distribution width ratio and risk of diabetic retinopathy among diabetes after PSM-1 | | | |
| --- | --- | --- | --- |
|  | Model 1  OR (95% CI), *P* | Model 2  OR (95% CI), *P* | Model 3  OR (95% CI), *P* |
| Hemoglobin-to-red cell distribution width ratio | 0.87 (0.78-0.97) 0.010 | 0.83 (0.73-0.95) 0.006 | 0.81 (0.69-0.93) 0.003 |
| Stratified by age |  |  |  |
| 20-44 years | 0.98 (0.69-1.39) 0.901 | 0.89 (0.52-1.52) 0.664 | 0.40 (0.13-1.27) 0.147 |
| 45-64 years | 0.92 (0.79-1.07) 0.286 | 0.90 (0.74-1.10) 0.299 | 0.88 (0.69-1.13) 0.322 |
| ≥65 years | 0.75 (0.63-0.89) 0.001 | 0.73 (0.61-0.89) 0.001 | 0.75 (0.60-0.93) 0.008 |
| Stratified by gender |  |  |  |
| male | 0.84 (0.71-0.99) 0.039 | 0.80 (0.65-0.98) 0.032 | 0.77 (0.60-0.99) 0.049 |
| female | 0.84 (0.71-0.99) 0.043 | 0.84 (0.70-1.01) 0.054 | 0.82 (0.68-0.99) 0.047 |
| Stratified by race |  |  |  |
| Non-Hispanic White | 0.79 (0.66-0.95) 0.013 | 0.73 (0.59-0.91) 0.006 | 0.69 (0.53-0.90) 0.007 |
| Non-Hispanic Black | 0.97 (0.82-1.16) 0.750 | 0.98 (0.82-1.17) 0.803 | 1.03 (0.84-1.27) 0.759 |
| Mexican American | 1.09 (0.91-1.31) 0.352 | 1.12 (0.91-1.38) 0.269 | 0.93 (0.73-1.19) 0.569 |
| Other | 0.80 (0.55-1.18) 0.264 | 0.77 (0.49-1.20) 0.253 | 0.94 (0.62-1.44) 0.780 |
| Stratified by PIR |  |  |  |
| <2 | 0.95 (0.83-1.09) 0.495 | 0.93 (0.79-1.09) 0.355 | 0.92 (0.77-1.10) 0.343 |
| ≥2 | 0.79 (0.66-0.94) 0.008 | 0.71 (0.56-0.91) 0.006 | 0.72 (0.54-0.95) 0.023 |
| Stratified by DBP |  |  |  |
| <90 mmHg | 0.90 (0.80-1.00) 0.059 | 0.86 (0.75-0.99) 0.037 | 0.85 (0.74-0.99) 0.041 |
| ≥90 mmHg | 0.84 (0.58-1.23) 0.383 | 0.86 (0.54-1.37) 0.537 | 1.07 (0.63-1.81) 0.800 |
| Stratified by HbA1c(%) |  |  |  |
| <7 | 0.81 (0.70-0.93) 0.003 | 0.77 (0.65-0.91) 0.002 | 0.81 (0.67-0.98) 0.030 |
| ≥7 | 0.86 (0.73-1.02) 0.089 | 0.79 (0.64-0.97) 0.025 | 0.80 (0.63-1.01) 0.067 |
| Stratified by age of diabetes onset |  |  |  |
| <30 years | 0.83 (0.70–0.98) 0.032 | 0.82 (0.69–0.97) 0.035 | 0.81 (0.67–0.98) 0.038 |
| ≥30 years | 0.84 (0.72–0.98) 0.041 | 0.83 (0.71–0.97) 0.043 | 0.82 (0.69–0.97) 0.046 |
| Stratified by Insulin usage |  |  |  |
| no | 0.82 (0.71–0.95) 0.034 | 0.81 (0.69–0.95) 0.037 | 0.80 (0.67–0.96) 0.040 |
| yes | 0.80 (0.67–0.95) 0.042 | 0.79 (0.66–0.94) 0.045 | 0.78 (0.65–0.94) 0.048 |
| Stratified by diabetes duration |  |  |  |
| <10 years | 1.04 (0.86-1.25) 0.712 | 1.00 (0.81-1.23) 0.997 | 0.94 (0.75-1.16) 0.549 |
| ≥10 years | 0.83 (0.72-0.96) 0.011 | 0.79 (0.66-0.95) 0.013 | 0.79 (0.65-0.96) 0.020 |
| Stratified by Smoking |  |  |  |
| no | 0.89 (0.76-1.05) 0.170 | 0.84 (0.68-1.04) 0.114 | 0.82 (0.66-1.03) 0.092 |
| yes | 0.85 (0.74-0.98) 0.022 | 0.82 (0.69-0.96) 0.014 | 0.81 (0.67-0.98) 0.031 |
| Stratified by Serum albumin |  |  |  |
| <3.5 g/dL | 1.98 (1.06-3.70) 0.044 | 1.98 (1.15-3.41) 0.022 | 1.78 (0.95-3.21) 0.069 |
| ≥3.5 g/dL | 0.86 (0.77-0.96) 0.008 | 0.80 (0.70-0.92) 0.001 | 0.79 (0.68-0.91) 0.001 |
| Stratified by UACR |  |  |  |
| <30 mg/g | 0.85 (0.74-0.98) 0.025 | 0.80 (0.68-0.95) 0.012 | 0.77 (0.64-0.93) 0.007 |
| 30-300 mg/g | 0.95 (0.78-1.16) 0.597 | 1.00 (0.79-1.26) 0.989 | 1.09 (0.82-1.44) 0.566 |
| >300 mg/g | 1.03 (0.78-1.37) 0.841 | 0.97 (0.65-1.44) 0.863 | 0.73 (0.43-1.24) 0.251 |
| Stratified by renal failure |  |  |  |
| no | 0.89 (0.80-1.00) 0.047 | 0.86 (0.75-0.98) 0.025 | 0.83 (0.71-0.96) 0.014 |
| yes | 0.60 (0.36-1.00) 0.057 | 0.59 (0.34-1.02) 0.065 | 0.63 (0.30-1.32) 0.229 |
| Abbreviations: PSM-1 refers to 1:1 propensity score matching based on age, sex, and race; OR, odds ratio; 95% CI, 95% confidence intenval; PIR: poverty income ratio; DBP, diastolic blood pressure; HbA1c, hemoglobin A1c; UACR, urinary albumin/creatinine ratio.  Mode 1 = Non-adiusted mode.  Mode 2 = Mode 1 + age, gender, and race were adusted.  Model 3 = Mode 2 + PIR, DBP, HbA1c(%) , diabetes duration, smoking, serum albumin, UACR and renal failure. The subgroup analysis was not adjusted for the stratification variable itself. | | | |

| **Table S7.** Associations between hemoglobin-to-red cell distribution width ratio and risk of diabetic retinopathy among diabetes after PSM-2 | | | |
| --- | --- | --- | --- |
|  | Model 1  OR (95% CI), *P* | Model 2  OR (95% CI), *P* | Model 3  OR (95% CI), *P* |
| Hemoglobin-to-red cell distribution width ratio | 0.88 (0.77-0.98) 0.022 | 0.85 (0.75–0.97) 0.013 | 0.83 (0.72–0.96) 0.009 |
| Stratified by age |  |  |  |
| 20-44 years | 0.97 (0.68–1.38) 0.875 | 0.88 (0.51–1.50) 0.640 | 0.42 (0.14–1.25) 0.140 |
| 45-64 years | 0.91 (0.78–1.06) 0.270 | 0.89 (0.73–1.09) 0.280 | 0.87 (0.68–1.12) 0.310 |
| ≥65 years | 0.76 (0.64–0.90) 0.002 | 0.74 (0.62–0.90) 0.002 | 0.76 (0.61–0.94) 0.009 |
| Stratified by gender |  |  |  |
| male | 0.85 (0.72–1.00) 0.042 | 0.81 (0.66–0.99) 0.035 | 0.78 (0.61–1.00) 0.051 |
| female | 0.83 (0.70–0.98) 0.040 | 0.83 (0.69–1.00) 0.050 | 0.81 (0.67–0.98) 0.045 |
| Stratified by race |  |  |  |
| Non-Hispanic White | 0.80 (0.67–0.96) 0.015 | 0.74 (0.60–0.92) 0.007 | 0.70 (0.54–0.91) 0.008 |
| Non-Hispanic Black | 0.96 (0.81–1.15) 0.730 | 0.97 (0.81–1.16) 0.780 | 1.02 (0.83–1.26) 0.740 |
| Mexican American | 1.08 (0.90–1.30) 0.340 | 1.11 (0.90–1.37) 0.260 | 0.94 (0.74–1.20) 0.580 |
| Other | 0.81 (0.56–1.19) 0.270 | 0.78 (0.50–1.21) 0.260 | 0.95 (0.63–1.45) 0.790 |
| Stratified by PIR |  |  |  |
| <2 | 0.94 (0.82–1.08) 0.480 | 0.92 (0.78–1.08) 0.340 | 0.91 (0.76–1.09) 0.330 |
| ≥2 | 0.80 (0.67–0.95) 0.009 | 0.72 (0.57–0.92) 0.007 | 0.73 (0.55–0.96) 0.024 |
| Stratified by DBP |  |  |  |
| <90 mmHg | 0.89 (0.79–1.00) 0.055 | 0.85 (0.74–0.98) 0.035 | 0.84 (0.73–0.98) 0.040 |
| ≥90 mmHg | 0.85 (0.59–1.24) 0.390 | 0.87 (0.55–1.38) 0.540 | 1.08 (0.64–1.82) 0.810 |
| Stratified by HbA1c(%) |  |  |  |
| <7 | 0.82 (0.71–0.94) 0.004 | 0.78 (0.66–0.92) 0.003 | 0.82 (0.68–0.99) 0.032 |
| ≥7 | 0.85 (0.72–1.01) 0.085 | 0.78 (0.63–0.96) 0.023 | 0.79 (0.62–1.00) 0.065 |
| Stratified by age of diabetes onset |  |  |  |
| <30 years | 0.84 (0.71–0.99) 0.034 | 0.83 (0.70–0.98) 0.037 | 0.82 (0.68–0.99) 0.040 |
| ≥30 years | 0.83 (0.71–0.97) 0.039 | 0.82 (0.70–0.96) 0.041 | 0.81 (0.68–0.96) 0.044 |
| Stratified by Insulin usage |  |  |  |
| no | 0.83 (0.72–0.96) 0.036 | 0.82 (0.70–0.96) 0.039 | 0.81 (0.68–0.97) 0.042 |
| yes | 0.81 (0.68–0.96) 0.044 | 0.80 (0.67–0.95) 0.047 | 0.79 (0.66–0.95) 0.050 |
| Stratified by diabetes duration |  |  |  |
| <10 years | 1.03 (0.85–1.24) 0.700 | 0.99 (0.80–1.22) 0.990 | 0.93 (0.74–1.15) 0.540 |
| ≥10 years | 0.84 (0.73–0.97) 0.012 | 0.80 (0.67–0.96) 0.014 | 0.80 (0.66–0.97) 0.021 |
| Stratified by Smoking |  |  |  |
| no | 0.88 (0.75–1.04) 0.160 | 0.83 (0.67–1.03) 0.110 | 0.81 (0.65–1.02) 0.090 |
| yes | 0.86 (0.75–0.99) 0.024 | 0.83 (0.70–0.97) 0.015 | 0.82 (0.68–0.99) 0.032 |
| Stratified by Serum albumin |  |  |  |
| <3.5 g/dL | 1.97 (1.05–3.68) 0.042 | 1.97 (1.14–3.40) 0.021 | 1.77 (0.94–3.20) 0.067 |
| ≥3.5 g/dL | 0.85 (0.76–0.95) 0.007 | 0.79 (0.69–0.91) 0.001 | 0.78 (0.67–0.90) 0.001 |
| Stratified by UACR |  |  |  |
| <30 mg/g | 0.84 (0.73–0.97) 0.023 | 0.79 (0.67–0.94) 0.011 | 0.76 (0.63–0.92) 0.006 |
| 30-300 mg/g | 0.94 (0.77–1.15) 0.590 | 0.99 (0.78–1.25) 0.980 | 1.08 (0.81–1.43) 0.560 |
| >300 mg/g | 1.02 (0.77–1.36) 0.830 | 0.96 (0.64–1.43) 0.850 | 0.72 (0.42–1.23) 0.240 |
| Stratified by renal failure |  |  |  |
| no | 0.88 (0.79–0.99) 0.045 | 0.85 (0.74–0.97) 0.023 | 0.82 (0.70–0.95) 0.013 |
| yes | 0.61 (0.37–1.01) 0.059 | 0.60 (0.35–1.03) 0.067 | 0.64 (0.31–1.33) 0.230 |
| Abbreviations: PSM-2 refers to 1:1 propensity score matching based on education level, marital status, BMI, alcohol intake, HDL-c, C-reactive protein, vitamin D, energy intake, and physical activity; OR, odds ratio; 95% CI, 95% confidence intenval; PIR: poverty income ratio; DBP, diastolic blood pressure; HbA1c, hemoglobin A1c; UACR, urinary albumin/creatinine ratio.  Mode 1 = Non-adiusted mode.  Mode 2 = Mode 1 + age, gender, and race were adusted.  Model 3 = Mode 2 + PIR, DBP, HbA1c(%) , diabetes duration, smoking, serum albumin, UACR and renal failure. The subgroup analysis was not adjusted for the stratification variable itself. | | | |

| **Table S8.** Potential mediating variables of hemoglobin-to-red cell distribution width ratio on diabetic retinopathy risk | | | | |
| --- | --- | --- | --- | --- |
| Variables | Mediation Effect | | | Proportion of mediation in total effect |
|  | Coefficient | 95% CI | P-Value |  |
| Poverty income ratio | -0.006 | -0.019, 0.019 | 0.229 | - |
| Diastolic blood pressure | -0.029 | -0.056, -0.008 | 0.013 | 15.9% |
| Serum albumin | 0.009 | -0.024, 0.042 | 0.605 | - |
| Hemoglobin A1c(%) | 0.051 | 0.012, 0.094 | 0.014 | -60.53% |
| Urinary albumin/creatinine ratio | 0.002 | -0.012, 0.019 | 0.721 | - |
| When exploring mediating variables, age diabetes diagnosed, age, gender, race, poverty income ratio, diastolic blood pressure, hemoglobin A1c(%) , diabetes duration, smoking, serum albumin, urinary albumin/creatinine ratio and renal failure are all adjusted (when exploring a certain variable, it is not adjusted). | | | | |
